# LC/MS Analysis of Tetrodotoxin and Its Deoxy Analogs in the Marine Puffer Fish *Fugu niphobles* from the Southern Coast of Korea, and in the Brackishwater Puffer Fishes *Tetraodon nigroviridis* and *Tetraodon biocellatus* from Southeast Asia

**DOI:** 10.3390/md8041049

**Published:** 2010-03-31

**Authors:** Jun-Ho Jang, Jong-Soo Lee, Mari Yotsu-Yamashita

**Affiliations:** 1 Graduate School of Agricultural Science, Tohoku University, 1-1 Tsutsumidori-Amamiyamachi, Aoba-ku, Sendai 981-8555, Japan; E-Mail: jun-ho-jang@uiowa.edu; 2 Department of Seafood Science and Technology, Institute of Marine Industry, Gyeongsang National University, Tongyeong, Gyeongnam 650-160, Korea; E-Mail: leejs@gnu.ac.kr

**Keywords:** tetrodotoxin, 6,11-dideoxytetrodotoxin, puffer fish, LC/MS, LC/MS/MS

## Abstract

Tetrodotoxin (TTX) and its deoxy analogs, 5-deoxyTTX, 11-deoxyTTX, 6,11-dideoxyTTX, and 5,6,11-trideoxyTTX, were quantified in the tissues of three female and three male specimens of the marine puffer fish, *Fugu niphobles,* from the southern coast of Korea, and in the whole body of the brackishwater puffer fishes, *Tetraodon nigroviridis* (12 specimens) and *Tetrodon biocellatus* (three specimens) from Southeast Asia using LC/MS in single ion mode (SIM). Identification of these four deoxy analogs in the ovarian tissue of *F. niphobles* were further confirmed by LC/MS/MS. TTX and 5,6,11-trideoxyTTX were detected in all three puffer fish species as the major TTX analogs, similar to Japanese *Fugu pardalis*. While 6,11-dideoxyTTX was also found to be a major analog in almost all tissues of Korean *F. niphobles*, this analog was minor in the two *Tetraodon* species and Japanese *F. pardalis*. Among the tissues of *F. niphobles*, the concentrations of TTXs were highest in the ovaries (female) and skin (female and male).

## 1. Introduction

**Figure 1:**
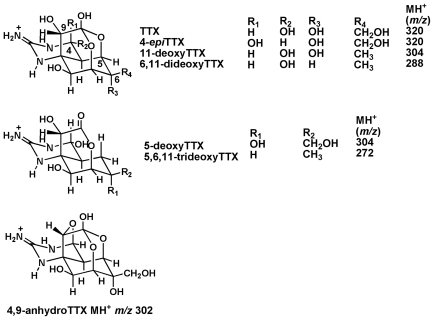
The chemical structures of TTX and its analogs found in puffer fishes.

Tetrodotoxin (TTX), the primary chemical toxin of puffer fishes, exists as a mixture of its analogs (Figure 1). Among them, 4-*epi*TTX and 4,9-anhydroTTX are the chemical equilibrium analogs of TTX [1,2]. Four deoxy analogs, such as 5-deoxyTTX [3], 11-deoxyTTX [4], 6,11-dideoxyTTX [5] and 5,6,11-trideoxyTTX [6], were also isolated from puffer fishes and/or newts as the chemical non-equilibrium analogs. The values of LD_50_ (50% lethal dose, mice, intraperitoneal injection) of TTX, 11-deoxyTTX, and 6,11-dideoxyTTX were determined as 10 μg/kg [7], 70 μg/kg [4], and 420 μg/kg [5], respectively, and the value of LD_99_ of 5,6,11-trideoxyTTX was 750 μg/kg [6]. The LD value of 5-deoxyTTX has not been determined yet.

We previously established the hydrophilic interaction liquid chromatography-electrospray ionization/mass spectrometry (HILIC-ESI/MS) system for TTXs [8,9]. This system has enabled us to quantify the four deoxy analogs of TTX which were not detectable with liquid chromatography fluorescent detection (LC-FLD) [8,11–12]. Using this LC/MS, distribution of TTX analogs among tissues of Japanese marine puffer fish *Fugu pardalis* was revealed, and 5,6,11-trideoxyTTX was found to be the major analog in all tissues [10]. Rodriguez *et al*. [13] also reported the presence of TTX and 5,6,11-trideoxyTTX as the major TTX analogs in the European trumpet shell, *Charonia lampas lampas*, using LC/MS/MS. Contrary to the marine animals, 5,6,11-trideoxyTTX was not detected in the newt, *Cynops ensicauda* [9]. It has not been clarified whether these deoxy analogs and TTX are chemically modified with each other in puffer fishes, or if they are all produced by the TTX producing bacteria [14,15]. However, these analogs can be speculated to be precursors or metabolites of TTX [16,17] because of their structural similarity. To support this assumption, we need to confirm that these deoxy analogs are commonly present in a range of species of puffer fishes.

Herein we examined the contents of TTX and its deoxy analogs in the tissues of three female and three male specimens of the marine puffer fish *F. niphobles* collected at the southern coast of Korea, and in two brackishwater puffer fishes *Tetraodon nigroviridis* and *Tetraodon biocellatus* from Southeast Asia, purchased in a pet market in Japan [18], by LC/MS in HILIC mode.

## 2. Results and Discussion

**Figure 2:**
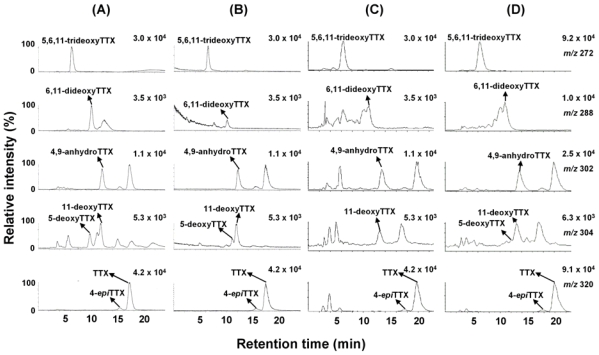
The mass chromatograms of LC/MS in SIM mode. (**A**) Volume of 4 μl of the semi-purified TTXs mixture containing TTX (53 pmol), 4-*epi*TTX (5.6 pmol), 4,9-anhydroTTX (7.4 pmol), 5-deoxyTTX (1.8 pmol), 11-deoxtyTTX (6.8 pmol), 6,11-dideoxyTTX (1.5 pmol), and 5,6,11-trideoxyTTX (38 pmol) prepared from ovary of *F. poecilonotus* as standard [10], (**B**) Sample solution (1 μl) of the ovary from female no. 3 *F. niphobles*, (**C**) Sample solution (1 μl) of whole body of no. 5 specimen of *T. nigroviridis,* (**D**) Sample solution (1 μl) of whole body of no. 2 specimen of *T. biocellatus*.

TTXs were quantified by LC/MS in single ion monitoring (SIM) mode, because the ratios of signal to noise of the peaks of TTXs were higher than those obtained by LC/MS/MS in multiple reaction monitoring (MRM) mode, probably due to some specific reasons related to our MS spectrometer. Tetrodonic acid was not analyzed in this study. In Figure 2, LC/MS separation in SIM mode of (B) the ovary from female no. 3 specimen of Korean *F. niphobles,* (C) whole body of no. 5 specimen of *T. nigroviridis,* and (D) whole body of no. 2 specimen of *T. biocellatus* from Southeast Asia are illustrated as representatives. Based on the peak area on the mass chromatograms, the concentrations of TTXs in the ovary from female no. 3 *F. niphobles* are estimated at TTX (263 nmol/g), 4-*epi*TTX (26 nmol/g), 4,9-anhydroTTX (83 nmol/g), 5-deoxyTTX (22 nmol/g), 11-deoxyTTX (39 nmol/g), 6,11-dideoxyTTX (310 nmol/g), and 5,6,11-trideoxyTTX (603 nmol/g). Similarly, the toxin contents in each tissue of three female and three male specimens of *F. niphobles,* and those in whole body of twelve specimens of *T. nigroviridis* and three specimens of *T. biocellatus* are determined, and summarized in Table 1 and Table 2, respectively.

**Figure 3:**
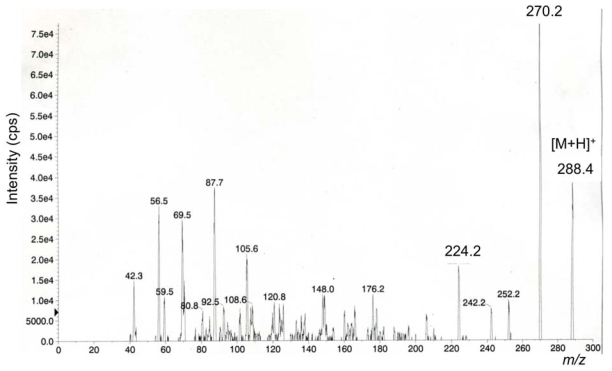
The fragment ion spectrum from [M+H]^+^ *m/z* 288 of 6,11-dideoxyTTX (150 pmol) injected into 0.2 mL/min of methanol. Collision energy: 42 eV.

**Figure 4:**
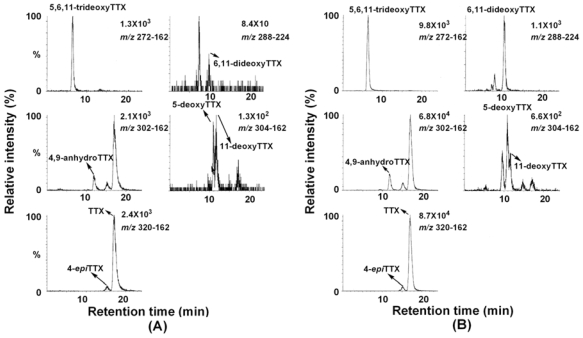
The mass chromatograms of the LC/MS/MS obtained under MRM operation (*m/z* 320–162 for TTX and 4-*epi*TTX, *m/z* 302–162 for 4,9-anhydroTTX, *m/z* 304–162 for 5-deoxyTTX and 11-deoxyTTX, 288–224 for 6,11-dideoxyTTX, and *m/z* 272–162 for 5,6,11-tridexyTTX). (A) Volume of 4 μl of the semi-purified TTXs mixture containing TTX (53 pmol), 4-*epi*TTX (5.6 pmol), 4,9-anhydroTTX (7.4 pmol), 5-deoxyTTX (1.8 pmol), 11-deoxtyTTX (6.8 pmol), 6,11-dideoxyTTX (1.5 pmol), and 5,6,11-trideoxyTTX (38 pmol) prepared from ovary of *F. poecilonotus* as standard [10], (B) Volume of 1 μl of ovary sample solution from female no. 3 *F. niphobles*.

We previously reported characteristic fragment ions, including a major ion at *m/z* 162, from [M+H]^+^ ions of TTX, 4-*epi*TTX, 4,9-anhydroTTX, 5-deoxyTTX, 11-deoxyTTX, and 5,6,11-trideoxyTTX by MS/MS (product ion) scan, and applied them to detection of TTXs by LC/MS/MS in MRM mode [8] as listed in Table 3. In the present study, we determined the major fragment ions by product ion scan from [M+H]^+^ *m/z* 288 for 6,11-dideoxyTTX for the first time (Figure 3). Distinctively from other TTX analogs [8], 6,11-dideoxyTTX produced the major fragment ion at *m/z* 224 instead at *m/z*162. Therefore, *m/z* 288–224 fragment was set up for detection of 6,11-dideoxyTTX by LC/MS/MS. Identification of TTX analogs by LC/MS in SIM mode was confirmed by LC/MS/MS in MRM mode, only for the ovary from female no. 3 Korean *F. niphobles* (Figure 4B).

As shown in Table 1 and Table 2, TTX and 5,6,11-trideoxyTTX are commonly detected as the major toxins in all tissues of Korean *F. niphobles* and also in the two *Tetraodon* species, similar to Japanese *Fugu pardalis* as we reported previously [10]. Interestingly, 6,11-dideoxyTTX, which was previously discovered in the ovaries of Japanese *F. pardalis* as a minor analog [5], is detected as another major TTX analog in almost all tissues in Korean *F. niphobles* with exception of the skin from females. Also, the concentration of 6,11-dideoxyTTX is higher than that of TTX in the intestine and liver from female no. 3 *F. niphobles*. This analog is also detected in some specimens of *T. nigroviridis* and *T. biocellatus* as a minor analog. 5-DeoxyTTX and 11-deoxyTTX are contained in low concentrations in almost all tissues of Korean *F. niphobles*, but they are less than detection limit (<0.5 nmol/g) in the muscle and testicle. 11-DeoxyTTX is a minor analog also in the two *Tetraodon* species, while 5-deoxyTTX is slightly detected only in *T. biocellatus* and it is less than detection limit (<0.5 nmol/g) in *T. nigroviridis.* Except for 5-deoxyTTX in *T. nigroviridis*, these data suggest that these four deoxy analogs of TTX are commonly present in puffer fishes, although the ratios of them are specific to the species of puffer fishes or to the regions where the fishes were collected.

Among the tissues of Korean *F. niphobles*, the concentration of TTXs is highest in the ovary and skin. This is in agreement with the report by Ryu *et al*. [19] that skin was highly toxic (10–674 mouse unit/g = 7–465 nmol/g TTX) in Korean *F. niphobles* by mouse assay method (1 mouse unit is equivalent to 220 ng of TTX). Also, this is consistent with the data reported by Kono *et al*. [20] that dietarily administrated TTX to Japanese cultured juvenile *F. niphobles* was first accumulated in liver and then transferred to skin. According to Ikeda *et al*. [21], the high toxicity in the ovaries of *Takifugu* (=*Fugu*) *poecilonotus* might account for the TTXs transfer from liver, skin, intestine, and muscle into the ovaries during spawning season. Unlike Korean *F. niphobles*, Japanese *F. pardalis* contained the highest concentration of TTXs in ovary and liver, and medium level TTXs in skin [10]. Edible parts except intestine and ovary of *F. niphobles,* which contained fewer than 10 mouse units per gram, are used in a favorite fish soup or other food materials in Korea and cause TTX poisoning sporadically. More careful precautions need to be taken to remove toxic body parts before cooking. Concerning the toxicity of the puffer fishes genus *Tetraodon*, Shin-Jung *et al*. [22] examined the toxicity of 42 and 12 specimens of *T. ocellatus* and *T. nigroviridis*, respectively, collected in Taiwan by using mouse bioassay, and identified TTX and 4,9-anhydroTTX in both species by LC. The authors reported the highest toxicity score of *T. nigroviridis* to be 124 mouse unit/g (=85 nmol/g TTX), rather lower than those found in our present study (Table 2).

## 3. Experimental Section

### 3.1. Puffer fishes specimens

Specimens of three female and three male each of *F. niphobles* (body weight 12–37 g) were collected on May 12^th^, 2008 at Tongyeong bay, South Korea. The twelve specimens of *T. nigroviridis* (body weight 0.8–3.8 g, sex not identified) and three specimens of *T. biocellatus* (body weight 1.6–3.7g, sex not identified) collected from Southeast Asia were purchased in a pet market of Miyagi Prefecture, Japan. Samples were immediately kept on ice, transported to the laboratory and kept frozen below −20 °C for no longer than a month until use.

### 3.2. Preparation of sample solutions

Sample solutions were prepared as we reported previously [8,9] with minor modification. *F. niphobles* were dissected to obtain organs of ovary/testis, liver, intestine, dorsal skin and dorsal muscle, while *T. nigroviridis* and *T. biocellatus* were used as whole body. The toxins were extracted from a part (1 g) of homogenized tissues with 5 mL of 0.05 M acetic acid (v/v) by heating in boiling water for 5 min. The obtained suspensions were centrifuged at 4,000 rpm for 15 min at 4 °C, then the supernatants were filled up to 5 mL with 0.05 M acetic acid. An aliquot (2 mL) was applied to the reverse phase cartridge column (Sep-Pak C18, Waters) which was equilibrated with water after washing with MeOH before use. The first passing solution (1.5 mL) was discarded, and the following 0.5 mL was collected and defatted with CHCl_3_ (0.5 mL). After removal of the remaining CHCl_3_ under the stream of N_2_ gas, the aqueous phase was lyophilized and filled up to 5 mL with water, and then loaded on an activated charcoal (0.5 mL), which was packed in a glass pipette. After washing the column with water (0.5 mL), the toxins were eluted with acetic acid/EtOH/H_2_O (1:50:49, 1.5 mL), and the volatile was evaporated from the eluate. The remaining residue was dissolved in 0.5 mL of 0.05 M acetic acid, and then ultrafiltered (Ultrafree-MC, 10,000 NMWL, Millipore, Bedford, MA) by centrifugation at 8,000 rpm for 15 min at 4 °C. This filtrate was used as the sample solution for LC/MS and LC/MS/MS analysis.

### 3.3. Preparation of semi-purified TTXs mixture as the standard for LC/MS

Semi-purified TTXs mixture was prepared from the ovary of *Fugu poecilonotus*, collected in Shimonoseki, Japan, by extraction of toxins with hot 0.05 M acetic acid, followed by partition with ethyl acetate and purification on a charcoal column [6]. TTX, 4-*epi*TTX, 4,9-anhydroTTX, 5-deoxyTTX, 11-deoxyTTX, 6,11-dideoxyTTX, and 5,6,11-trideoxyTTX in the obtained mixture were quantified using LC/MS in SIM mode on the basis of the standard curve for each analog drawn using highly purified analogs [8–10]. Because highly purified TTX analogs are very limited, we used this semi-purified TTXs mixture as the standard for LC/MS.

### 3.4. LC/MS, MS/MS, and LC/MS/MS

LC/MS was performed based on HILIC as we reported previously [9, 10]. Briefly, LC system consisted of a Shimadzu LC-10AD pump (Japan) and a TSKgel Amide-80 column (2.0-i.d. x 150 mm, 5 μm, Toso, Tokyo, Japan). LC was performed using an aqueous solution containing 16 mM ammonium formate buffer (pH 5.5) and acetonitrile (3:7, v/v) as a mobile phase at a flow rate of 0.2 mL/min at 25 °C. LC/MS, LC/MS/MS and flow-injection MS/MS experiments were recorded on API2000 mass spectrometer (Applied Bio-systems MDS SCIEX, Foster City, CA) equipped with an ESI source in the positive-ion mode. ESI was evoked by a spray voltage of +5.5 kV and heated capillary temperature was maintained at 500 °C. Six ions at *m/z* 272, 288, 302, 304 and 320 corresponding to the [M+H]^+^ ions of TTX analogs were detected in SIM mode, respectively. The dwell time was set at 200 ms per Da. The MS/MS measurements of 6,11-dideoxyTTX was based on collision induced dissociation (CID) occurring in the collision cell (Q2) of the triple quadrupole at a collision energy of 42 eV. Nitrogen was used as the target gas. 150 pmol of authentic 6,11-dideoxyTTX [5] (4 μl in 0.05M acetic acid) was introduced into the mass spectrometer by flow injection with methanol at a flow rate of 0.2 mL/min. The LC/MS/MS was performed under the same conditions as those used for the LC/MS and MS/MS experiments. The ions *m/z* 320–162 for TTX and 4-*epi*TTX, *m/z* 302–162 for 4,9-anhydroTTX, *m/z* 304–162 for 5-deoxyTTX and 11-deoxyTTX, *m/z* 288–224 for 6,11-dideoxyTTX, and *m/z* 272–162 for 5,6,11-tridexyTTX were detected in MRM mode [8]. The minimum TTX and its analogs to be detected were 0.5 nmol/g tissue in SIM mode.

## 4. Conclusions

In this study, four deoxy analogs of TTX were all detected in three species of puffer fishes tested with exception of 5-deoxyTTX in *T. nigroviridis*. We also found these deoxy analogs in ovary from Japanese *Fugu poecilonotus* when we prepared standard mixture of TTXs for LC/MS as described in the Experimental section. These results indicate that the deoxy analogs of TTX are common analogs in a range of puffer fishes. For the next work, we should investigate whether these deoxy analogs are chemically modified with each other in puffer fishes, or if they are provided from external origin, such as bacteria [14,15] and then accumulated in puffer fishes. The ability of puffer fishes to accumulate TTX was confirmed by Yamamori et al.[23] and Kono et al.[20] by the fact that cultured juvenile non-toxic Japanese *Takifugu* (*Fugu*) *niphobles* become toxic when TTX and its analogs were dietarily administered. Kono *et al*.[24] also reported that cultured juvenile *F. niphobles* administered TTX by intramuscular injection did not transform TTX to deoxy analogs or to 11-oxoTTX (which has CHO in the position of R_4_ in Figure 1 instead of CH_2_OH of TTX). However, transformation from deoxy analogs or 11-oxoTTX to TTX has not been tested, and dietary administration might be important for such experiments. We will further examine whether these deoxy analogs are the products of transformation or metabolites in puffer fishes.

## Figures and Tables

**Table 1:** Anatomical distribution of TTX and its analogs in *F. niphobles* (n = 3).

Tissue	Sex	Toxin content (nmol/g)						
		TTX	4-*epi*TTX	4,9-anhydro TTX	5-deoxy TTX	11-deoxy TTX	6,11-dideoxy TTX	5,6,11-trideoxy TTX
Ovary	F	129–263	10–26	30–83	4–22	8–39	77–310	289–603
Testicle	M	1–11	<0.5–1	<0.5–3	<0.5	<0.5	<0.5–9	1–13
Liver	F	4–24	<0.5–6	5–39	<0.5–3	<0.5–1	1–24	3–16
	M	9–31	1–3	9–15	<0.5–2	<0.5–1	<0.5–59	10–18
Skin	F	78–93	8–10	21–30	3–10	3–8	6–13	110–155
	M	38–197	2–9	6–26	1–7	1–9	2–28	39–153
Muscle	F	3–8	<0.5–1	<0.5–1	<0.5	<0.5	1–6	5–7
	M	8–16	1–3	<0.5–1	<0.5	<0.5	<0.5–5	<0.5–14
Intestine	F	8–67	1–16	7–110	1–4	<0.5–12	11–145	25–71
	M	3–7	<0.5–1	2–6	<0.5	<0.5	2–28	5–7

**Table 2:** The contents of TTX and its analogs in the whole body extracts of *T. nigroviridis* (n = 12) and *T. biocellatus* (n = 3).

Species	Toxin content (nmol/g)						
	TTX	4-*epi*TTX	4,9-anhydro TTX	5-deoxy TTX	11-deoxy TTX	6,11-dideoxy TTX	5,6,11-trideoxy TTX
*T. nigroviridis*	0.6–294	<0.5–5	<0.5–47	<0.5–7	<0.5–7	<0.5–8	<0.5–151
*T. biocellatus*	27–190	2–16	4–41	<0.5–2	<0.5–17	<0.5–18	21–202

**Table 3:** MRM conditions for TTX analogs in positive ESI-mode. Collision Energy: 42 eV.

	Precursor (*m/z*)	Product (*m/z*)	Retention time (min)
TTX	320	162	17.04
4-*epi*TTX	320	162	15.15
4,9-anhydroTTX	302	162	11.75
5-deoxyTTX	304	162	10.82
11-deoxyTTX	304	162	11.50
6,11-dideoxyTTX	288	224	10.04
5,6,11-trideoxyTTX	272	162	6.24

## References

[1] Goto T Kishi Y Takahashi S Hirata Y Tetrodotoxin Tetrahedron 1965 21 2059 2088 5896483 10.1016/s0040-4020(01)98344-9

[2] Nakamura M Yasumoto T Tetrodotoxin derivatives in puffer fish Toxicon 1985 23 271 276 4024136 10.1016/0041-0101(85)90149-7

[3] Yotsu-Yamashita M Schimmele B Yasumoto T Isolation and structural assignment of 5-deoxytetrodotoxin from the puffer fish *Fugu poecilonotus* Biosci Biotechnol Biochem 1999 63 961 963 10380642 10.1271/bbb.63.961

[4] Yasumoto T Yotsu M Murata M Naoki H New tetrodotoxin analogue from the newt *Cynops ensicauda* J Am Chem Soc 1988 110 2344 2345

[5] Jang JH Yotsu-Yamashita M 6,11-DideoxyTTX from the puffer fish, *Fugu pardalis* Toxicon 2007 50 947 951 17826815 10.1016/j.toxicon.2007.06.026

[6] Yotsu-Yamashita M Yamagishi Y Yasumoto T 5,6,11-Trideoxytetrodotoxin from the puffer fish, *Fugu poecilonotus* Tetrahedron Lett 1995 36 9329 9332

[7] Kao CY Furman F A Pharmacological studies on tetrodotoxin, a potent neurotoxin J Pharmacol Exp Ther 1963 140 31 40 13962288

[8] Shoji Y Yotsu-Yamashita M Miyazawa T Yasumoto T Electrospray ionization mass spectrometry of tetrodotoxin and its analogs: liquid mass spectrometry, and liquid chromatography/tandem mass spectrometry Anal Biochem 2001 290 10 17 11180932 10.1006/abio.2000.4953

[9] Nakagawa T Jang J Yotsu-Yamashita M Hydrophilic interaction liquid chromatography-electrospray ionization mass spectrometry of tetrodotoxin and its analogs Anal Biochem 2006 352 142 144 16574054 10.1016/j.ab.2006.02.010

[10] Jang J Yotsu-Yamashita M Distribution of tetrodotoxin, saxitoxin, and their analogs among tissues of the puffer fish *Fugu pardalis* Toxicon 2006 48 980 987 16997342 10.1016/j.toxicon.2006.07.034

[11] Yasumoto T Michishita T Fluorometric determination of tetrodotoxin by high performance liquid chromatography Agric Biol Chem 1985 49 3077 3080

[12] Yotsu M Endo A Yasumoto T An improved tetrodotoxin analyzer Agric Biol Chem 1989 53 895 898

[13] Rodriguez P Alfonso A Vale C Alfonso C Vale P Tellez A Botana LM First toxicity report of tetrodotoxin and 5,6,11-trideoxyTTX in the trumpet shell *Charonia lampas lampas* in Europe Anal Chem 2008 80 5622 5629 18558725 10.1021/ac800769e

[14] Yasumoto T Yasumura D Yotsu M Michishita T Endo A Kotaki Y Bacterial production of tetrodotoxin and anhydrotetrodotoxin Agric Biol Chem 1986 50 793 795

[15] Noguchi T Jeo J Arakawa O Sugita H Deguchi Y Shida Y Hashimoto K Occurrence of tetrodotoxin and anhydrotetrodotoxin in *Vibrio* sp. Isolated from the intestines of a xanthid crab, *Atergatis floridus* J Biochem 1986 99 311 314 3754255 10.1093/oxfordjournals.jbchem.a135476

[16] Yasumoto T Yotsu-Yamashita M Chemical and etiological studies on tetrodotoxin and its analogs J Toxicol Toxin Rev 1996 15 81 90

[17] Yotsu-Yamashita M Chemistry of puffer fish toxin J Toxicol Toxin Rev 2001 20 51 66

[18] Jang JH Yotsu-Yamashita M Tetrodotoxin and 5,6,11-trideoxytetrodotoxin in *Tetraodon nigroviridis* and *T. biocellatus* collected from Southeast Asia Proceedings of the 5th World Fisheries Congress, Preliminary data of Tetraodon nigroviridis and Tetraodon biocellatus has been reported in the proceedings Yokohama, Japan 20–24 October 2008

[19] Ryu CH Kim DG Kim JH Jang JH Lee JS Toxicity of the Grass puffer, *Takifugu niphobles* (Bogseom) J Korean Soc Food Sci Nutr 2003 32 986 990 (in Korean)

[20] Kono M Matsui T Furukawa K Yotsu-Yamashita M Yamamori K Accumulation of tetrodotoxin and 4,9-anhydrotetrodotoxin in cultured juvenile kusafugu *Fugu niphobles* by dietary administration of natural toxic komonfugu *Fugu poecilonotus* liver Toxicon 2008 51 1269 1273 18420245 10.1016/j.toxicon.2008.02.017

[21] Ikeda K Emoto Y Tatsuno R Wang JJ Ngy L Taniyama S Takatani T Arakawa O Maturation-associated changes in toxicity of the pufferfish *Takifugu poecilonotus* Toxicon 2010 55 289 297 19682483 10.1016/j.toxicon.2009.08.001

[22] Shin-Jung L Liao CF Arakawa O Noguchi T Hwang DF Toxicities of two freshwater puffers in Taiwan J Nat Toxins 2002 11 103 110 12009110

[23] Yamamori K Furukawa K Kono M Matsui T The toxification of juvenile cultured kusafugu *Takifugu niphobles* by oral administration of crystalline tetrodotoxin J Food Hyg Soc Jpn 2004 45 73 75 (in Japanese) 10.3358/shokueishi.45.73 15272603

[24] Kono M Matsui T Furukawa K Takase T Yamamori K Kaneda H Aoki D Jang JH Yotsu-Yamashita M Examination of transformation among tetrodotoxin and its analogs in the living cultured juvenile puffer fish, kusafugu, *Fugu niphobles* by intramuscular administration Toxicon 2008 52 714 720 18775741 10.1016/j.toxicon.2008.08.002

